# EnanDIM - a novel family of L-nucleotide-protected TLR9 agonists for cancer immunotherapy

**DOI:** 10.1186/s40425-018-0470-3

**Published:** 2019-01-08

**Authors:** Kerstin Kapp, Barbara Volz, Michael A. Curran, Detlef Oswald, Burghardt Wittig, Manuel Schmidt

**Affiliations:** 1 Department of Early & Translational R&D, Mologen AG, Fabeckstr. 30, 14195 Berlin, Germany; 2 Department of Immunology, The University of Texas MD Anderson Cancer Center, Houston, USA; 3 Scientific advisor to Mologen AG, Berlin, Germany

**Keywords:** TLR9 agonist, EnanDIM®, Cancer immunotherapy, Tumor microenvironment, IFN-alpha, T cell response

## Abstract

**Background:**

Toll-like receptor 9 agonists are potent activators of the immune system. Their clinical potential in immunotherapy against metastatic cancers is being evaluated across a number of clinical trials. TLR9 agonists are DNA-based molecules that contain several non-methylated CG-motifs for TLR9 recognition. Chemical modifications of DNA backbones are usually employed to prevent degradation by nucleases. These, however, can promote undesirable off-target effects and therapeutic restrictions.

**Methods:**

Within the EnanDIM® family members of TLR9 agonists described here, D-deoxyribose nucleotides at the nuclease-accessible 3′-ends are replaced by nuclease-resistant L-deoxyribose nucleotides. EnanDIM® molecules with varying sequences were screened for their activation of human peripheral blood mononuclear cells based on secretion of IFN-alpha and IP-10 as well as activation of immune cells. Selected molecules were evaluated in mice in a maximum feasible dose study and for analysis of immune activation. The ability to modulate the tumor-microenvironment and anti-tumor responses after EnanDIM® administration was analyzed in syngeneic murine tumor models.

**Results:**

The presence of L-deoxyribose containing nucleotides at their 3′-ends is sufficient to prevent EnanDIM® molecules from nucleolytic degradation. EnanDIM® molecules show broad immune activation targeting specific components of both the innate and adaptive immune systems. Activation was strictly dependent on the presence of CG-motifs, known to be recognized by TLR9. The absence of off-target effects may enable a wide therapeutic window. This advantageous anti-tumoral immune profile also promotes increased T cell infiltration into CT26 colon carcinoma tumors, which translates into reduced tumor growth. EnanDIM® molecules also drove regression of multiple other murine syngeneic tumors including MC38 colon carcinoma, B16 melanoma, A20 lymphoma, and EMT-6 breast cancer. In A20 and EMT-6, EnanDIM® immunotherapy cured a majority of mice and established persistent anti-tumor immune memory as evidenced by the complete immunity of these mice to subsequent tumor re**-**challenge.

**Conclusions:**

In summary, EnanDIM® comprise a novel family of TLR9 agonists that facilitate an efficacious activation of both innate and adaptive immunity. Their proven potential in onco-immunotherapy, as shown by cytotoxic activity, beneficial modulation of the tumor microenvironment, inhibition of tumor growth, and induction of long-lasting, tumor-specific memory, supports EnanDIM® molecules for further preclinical and clinical development.

**Electronic supplementary material:**

The online version of this article (10.1186/s40425-018-0470-3) contains supplementary material, which is available to authorized users.

## Background

Toll-like receptors (TLR) belong to the group of pattern recognition receptors (PRR) that identify pathogen-associated molecular patterns (PAMP), which are ubiquitously presented by pathogens but are essentially absent in vertebrates. TLR enable immune cells to fight pathogens by first activating the innate immune response, followed by the induction of antigen-specific effector- as well as memory T cells of adaptive immunity. Therefore, TLR agonists are attractive candidates for the development of therapeutic immune modulators to treat a broad range of diseases like cancer, asthma, allergies, or infections [1–3].

Among the more than ten currently known TLR in humans, TLR9 is predominantly expressed by plasmacytoid dendritic cells (pDC) and B cells and plays a major role in detecting invading pathogens with subsequent activation of the immune system [4, 5]. TLR9 recognizes non-methylated CG-motifs as PAMP, which are predominantly present in pathogenic DNA, but underrepresented in human nuclear DNA. Since TLR9 is known to broadly activate both the innate and adaptive immunity, TLR9-triggered immune activation can re-activate immune surveillance to effectively recognize tumor-specific antigens on cancer cells of tumor patients. For immunotherapeutic approaches PAMP can be mimicked by synthetic oligodeoxynucleotides (ODN) containing non-methylated CG-motifs [6, 7].

There is already a remarkable history of synthetic ODN developed to target TLR9 dating back to the first synthetic DNA oligonucleotide under clinical investigation which was the chemically-modified linear CPG-7909 (PF-3512676, ProMune) [8]. Other TLR9 agonists with a similar chemical composition soon followed suit especially for application to cancer treatment [2, 7, 9]. As linear, single-stranded CpG-ODN with natural phosphodiester (PO) backbones are prone to degradation by nucleases, protective modifications are necessary to ensure their persistence in vivo. Therefore, it is common to chemically modify these CpG-ODN with phosphorothioates (PTO) as previously used for antisense therapeutics [10]. However, these PTO-modifications lead to off-target side effects like prolongation of blood clotting time via inhibition of the intrinsic tenase complex [11, 12], non-specific binding to various proteins (i.e., transcription factors), thereby affecting cell signaling [13], platelet activation [14] and causing acute toxicities via complement activation in rhesus monkeys [15, 16]. In mice, these chemical modifications dramatically altered morphology and functionality of lymphoid organs, and can induce hemophagocytic lymphohistiocytosis and macrophage activation syndrome [17–19]. Furthermore, the resulting narrow therapeutic window of the early PTO-modified TLR9 agonists led to a discontinuation of advanced clinical studies [20, 21].

A second molecular family of TLR9 agonists, dSLIM®, was recently introduced which consists of dumbbell-shaped, covalently-closed DNA molecules devoid of any PTO or other artificial modifications [19, 22].

Here, we describe the development of EnanDIM® molecules which constitute a novel molecular family of L-nucleotide-protected TLR9 agonists. The members of the EnanDIM® family described here are stabilized and protected against nucleolytic degradation through enantiomeric nucleotides by positioning of L-deoxyribose-containing nucleotides at the DNA 3′-end. Although not prevalent in current vertebrates L-deoxyribose-containing nucleotides are capable of forming L-DNA, the enantiomer of natural D-DNA [23]. As DNA processing enzymes, like nucleases, and DNA components co-evolved, present mammalian exonucleases are blind for L-deoxyribose and the resulting L-DNA backbone thereby leaving L-nucleotide-protected ODN intact [24, 25]. Here, we investigated the EnanDIM® molecular family with respect to immunological potential – both in vitro and in vivo – and assayed for possible toxicological effects of maximum feasible doses in mice. To establish its potential in immuno-oncology, we evaluated the capacity of EnanDIM® molecules to modulate the tumor microenvironment (TME) and characterized the resulting anti-tumor effects, including long-term immune memory in various mouse tumor models.

The necessary and sufficient properties as immune surveillance reactivators (ISR) for cancer immunotherapy, i.e. efficacious and broad activation of innate and adaptive immunity, absence of clinically relevant off-target effects, and stability against nucleolytic degradation, are all successfully realized in EnanDIM®, the novel molecular family of TLR9 agonists.

## Methods

### L-nucleotide-protected TLR9 agonists

ODN with terminal L-nucleotides were synthesized by BioSpring, Axolabs or TIB Molbiol. After chromatographic purification, ODN were either ultra-diafiltrated or reconstituted in the indicated solvent.

### In vitro stimulation of cells

Buffy coats from anonymized healthy donors were obtained from the “DRK-Blutspendedienst - Ost”. Peripheral blood mononuclear cells (PBMC) were isolated by density gradient centrifugation using Ficoll (Biochrom). pDC were prepared using the Human Diamond pDC Isolation Kit (Miltenyi Biotec) according to the manufacturer’s instructions. Cells were cultured in complete medium (RPMI1640 [Lonza] with 2 mM UltraGlutamine [Lonza] supplemented with 10% [*v*/v] fetal calf serum [Linaris], 100 U/ml Penicillin and 100 μg/ml Streptomycin [Lonza]) in flat-bottom plates (PBMC, 6 million cells/ml) or 96-well round bottom plates (pDC, 0.25 million cells/ml, in the presence of 10 ng/ml recombinant IL(interleukin)-3 [PeproTech]). B18R (eBioscience), a vaccinia virus-encoded receptor with specificity to type I interferons, was used at a final concentration of 0.5 μg/ml.

### Flow cytometry

Cells were surface-stained with monoclonal antibodies in PBS containing 10% (*v*/v) human serum, 2.5% (v/v) FBS and 0.1% (*w*/*v*) azide on ice. The following antibodies were used: anti-lineage cocktail 1, anti-CD123 (7G3), anti-HLA-DR (L243), anti-CD40 (5C3), anti-CD11c (B-ly6), anti-CD86 (2331, FUN-1), anti-CD19 (4G7), anti-CD69 (FN50), all from BD Biosciences and anti-CD14 (61D3), anti-CD169 (7–239), anti-CD3 (OKT-3), anti-CD56 (MEM188), anti-CD80 (2D10), all from eBioscience.

All flow cytometric parameters of cells were acquired on a FACSCalibur (BD Biosciences). Frequencies were related to the indicated parent populations; geometric means of fluorescent cells were indicated as mean fluorescence intensity (MFI). Data were analyzed with the FlowJo software.

### Cytokine and chemokine determination

Secreted cytokines were accumulated in cell growth medium for 2d. ELISA for IFN-alpha (eBioscience), IFN-gamma (OptEIA Human IFN gamma ELISA Set, BD Biosciences), IP-10 (interferon-inducible protein 10, CXCL10), IL-8, and MCP-1 (monocyte chemoattractant protein-1, CCL2) (all from R&D Systems) were performed in duplicates according to the manufacturer’s instructions. Optical density was measured at 450 nm; the data were analyzed with the MicroWin software (Berthold Technologies). Alternatively, cytokine levels in the cell growth medium were determined in duplicates by a bead-based multiplex immunoassay (FlowCytomix from eBioscience) according to the manufacturer’s instructions. Data were acquired on a FACSCalibur and evaluated with the FlowCytomixPro software (eBioscience).

### Cytotoxicity assay

Jurkat cells (DSMZ) were used as target cells and labelled for 5 min at 37 °C in RPMI1640 (Lonza) with the lipophilic dye DiI (Invitrogen/Life Technologies) at a final concentration of 1 μM. After 18 h, 50,000 target cells were co-cultured with effector cells (treated PBMC) in three different ratios (effector:target 20:1, 10:1, and 5:1). For positive control, PBMC were cultured in 2000 U/ml IL-2 (PeproTech). Cultures were performed in complete medium and were run for 4 h at 37 °C in 96-well round bottom plates in duplicates. Subsequently, cells were stained with 7-AAD (BD Biosciences). On a FACSCalibur (BD Biosciences) 5000 target cells were acquired. The percentage of dead target cells was determined from 7-AAD-positive cells and specific cytotoxicity calculated: Specific cytotoxicity (%) = ((% 7-AAD + of Dil + induced)-(% 7-AAD + of Dil + spontaneous))/(100% - % 7-AAD+ of Dil + spontaneous)*100.

### In vitro TLR9 model

A murine reporter cell line (ELAM41) was obtained from K. Stacey [26]. Briefly, ELAM41 was generated by stably transfecting cells from the established mouse macrophage line RAW264.7 with a fluorescent protein-expressing DNA construct under the control of the human nuclear factor kappa-light-chain-enhancer of activated B-cells (NF-kappaB) responsive elastin promoter [26]. Thereby, the endogenous mouse TLR9 of ELAM41 cells was functionally coupled to the expression of the enhanced green fluorescent protein (eGFP). ELAM41 cells were incubated in the presence of the indicated concentrations of EnanDIM-C or the CG-free variant of EnanDIM-C, EnanDIM-C(-CG). If not shown otherwise, after 7 h the amount of fluorescent protein was determined via flow cytometry.

### Maximum feasible dose (MFD) and immunological study

CD-1 mice were subcutaneously (s.c.) injected with vehicle (0.9% NaCl), or a total dose of 10 mg EnanDIM-C or 50 mg EnanDIM-A after being assigned to experimental groups with each 10 mice by the body weight stratification method. The total dose was divided into four different injections of 0.25 mL per animal, each two hours apart, administered to four different sites on the back on day 1 of the study. Safety assessment relied on observed mortality, clinical signs and body weight recorded throughout the study period (15d). Immune response was assessed by the determination of CD169-positive cells within the CD11b^+^CD11c^−^ monocyte/macrophage population (via flow cytometry, the following antibodies were used: anti-CD169, clone SER-4 [eBioscience]; anti-CD11b, clone M1/70 [BD Biosciences]; anti-CD11c, clone HL3 [BD Biosciences]) and analysis of IP-10 (via ELISA, R&D Systems) at two time points: 24 h after first injection and at sacrifice (day 15). All animals were sacrificed and subjected to a gross necropsy consisting of a macroscopic evaluation of the tissues/organs contained in the abdominal and thoracic cavities.

To determine the immunological profile after a single administration EnanDIM-C/−A each 9 female Balb/c mice were distributed into 5 experimental groups by body weight. Animals were injected s.c. with vehicle (PBS), 200 μg or 1000 μg of either EnanDIM-C or EnanDIM-A, respectively. Three animals from each group were sacrificed at different time points: 6 h, 12 h and 24 h after injection. An additional group of three non-injected mice served as naïve control (time point 0 h). IP-10 levels were determined as above.

### Mouse tumor models

Female C57BL/6 and Balb/c mice (age 6–8 weeks) were housed and treated in accordance with the regulations of the Association for Assessment and Accreditation of Laboratory Animal Care (AAALAC). Tumors were engrafted by s.c. injection of 100 μl tumor cell suspension in the flank. Tumor length and width were determined using calipers and volume was calculated as (width^2^ × length)/2. Mice were randomized prior to i.tu. treatment when tumors were well established (40–140 mm^3^, reached at day 3 to 13 after tumor inoculation), mice being treated with s.c. application of EnanDIM were randomized according to body weight and treatment was started the day after tumor inoculation.

*Tumor growth evaluation*. The following tumor cells were used: MC38 (1 × 10^6^ cells), B16F10 (2 × 10^5^ cells), Pan02 (3 × 10^6^ cells), CT26 (1-3 × 10^5^), EMT-6 (5 × 10^5^ cells), and A20 (5 × 10^5^ cells). 50 μl EnanDIM-C (250 μg, dissolved in 1 mM KCl, 5% glucose) or vehicle was given intratumorally (i.tu.) three times per week for 3 weeks. For s.c. administrations in the CT26 model, the same dosing scheme was used. Animals were sacrificed when tumor size exceeded a pre-determined endpoint. For re-challenge experiments surviving mice were re-inoculated with the same kind and number of tumor cells into the opposite flank, but without further treatment. Naïve mice were inoculated with tumor cells without any treatment as re-challenge controls.

*ELISpot assay.* Spleen cells of 8 mice surviving double EMT-6 tumor inoculation as well as CT26 tumor inoculation and spleen cells from three naïve mice were prepared. For ELISpot assay (Mabtech, No. 3321-4HPW-2) 8 × 10^5^ spleen cells were co-cultured with 8 × 10^4^ mitomycin C-treated (100 μg/ml) tumor cells (EMT-6, CT26, Renca) or with AH1 peptide (Anaspec, 1 μg/ml, H2-Ld-restricted epitope derived from glycoprotein 70 expressed in CT26 cells) for 24 h in triplicates. Detection of IFN-gamma secreting cells were done according to the instructions of the manufacturer. For positive controls spleen cells were incubated with 500 ng/ml PMA plus 1 μg/ml Ionomycin; for negative controls, spleen cells were cultured without any additives. Number of spots was analyzed in an ELISpot reader (AID *i*Spot). For analysis number of spots in the “splenocytes only” approach was subtracted from the respective approaches with tumor cells /tumor peptides.

*Immunohistochemical analysis of tumors*. Tumor inoculation was done with 5 × 10^4^ CT26 cells (+ 50% matrigel). Ten days later, 50 μl of EnanDIM-C (200 μg in 1 mM KCl, 5% glucose) or vehicle were applied i.tu.. After four injections over 7 days the tumors were sampled and subjected to immunohistochemistry (IHC). Tumors were embedded in Tissue-TEK OCT (Sakura Finetek Inc.) and stored at − 20 °C. 5 μm cryosections were prepared, fixed in acetone and stained using the Ventana Discovery XT system (Roche). Sections were incubated with Rat anti-mouse CD8alpha (clone 53–6.7, eBioscience) at a dilution 1:500 (*v*/v) for 60 min. Thereafter they were incubated with Rabbit anti-rat IgG Fc antibodies (clone R18–2, Abcam) at a dilution 1:500 (v/v) for 32 min followed by a detection using omniMap anti-RabbitHRP (Roche) for 16 min. The chromoMap DAB kit (Roche) was used as substrate for visualization. Counterstaining was performed with hematoxylin. Slides were scanned (NanoZoomer, Hamamatsu) and tumor center and margin were manually scored by two experienced operators in a blinded fashion (1: no labeling, 2: few labeling, 3: intermediate labeling, 4 = intense labeling)*.*

### Statistical analyses

Data were analyzed with GraphPad Prism 7 (GraphPad Software Inc.). *P* values < 0.05 were considered significant. The statistical analyses are specified in the figure legends.

## Results

### Design of L-nucleotide-protected TLR9 agonists without chemical modification

**Fig. 1:**
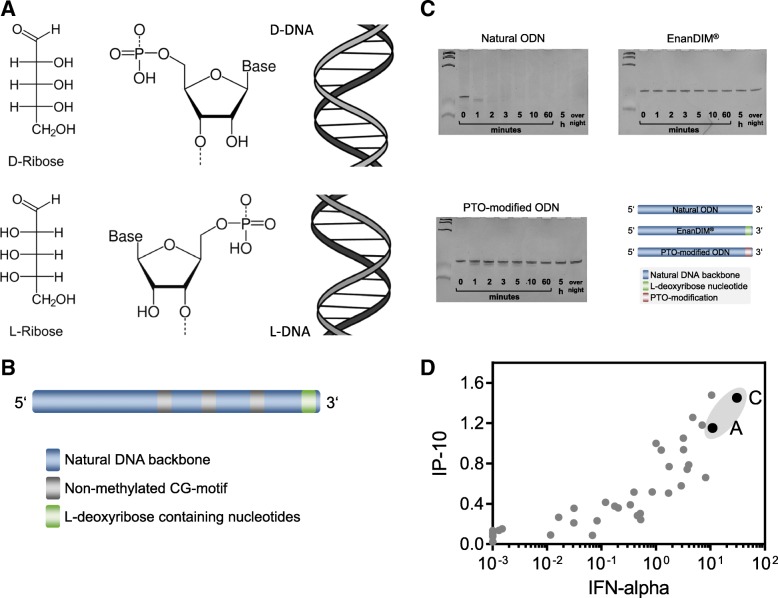
Enantiomeric nucleotides as basis for EnanDIM® and their broad immune surveillance reactivation. **a** Nucleotides derived from D-ribose (top) and from L-ribose (bottom). **b**, schematic structure of linear EnanDIM® with their key structural components. **c**, time course of stability against exonuclease: Natural, or PTO-modified ODN, or L-nucleotide-protected EnanDIM® were incubated with T7 DNA-polymerase in the absence of NTP for the indicated times. Samples were subjected to gel electrophoresis (24% acrylamide), and DNA was visualized by ethidiumbromide. **d**, screening for IP-10 and IFN-alpha production: incubation of human PBMC with various EnanDIM® molecules differing in their nucleotide sequence as well as a reference molecule at a final concentration of 3 μM for 48 h in vitro. IP-10 and IFN-alpha values after stimulation with EnanDIM were normalized to the reference molecule (means from 3 to 26 different molecules): EnanDIM-A/-C are shown as black solid circles

In contrast to CpG-ODN which achieve metabolic stability mainly by chemical modifications to its backbone, the new family of DNA-based immunomodulators, EnanDIM®, is protected from degradation by a different approach. The here described linear ODN for TLR9 activation are protected against 3′-exonucleolytic degradation by the presence of L-deoxyribose containing nucleotides at their 3′-ends (Fig. 1a, b). Exonucleases and other DNA processing enzymes recognize D-nucleotides and are blind to L-nucleotides, thereby rendering the 3′-end “incognito” to degradation processes including, for example, the exonuclease-activity of T7 polymerase (Fig. 1c).

The DNA sequence of the members of this L-nucleotide-protected ODN family was optimized in a screening system using incubation with PBMC. The key optimization parameters for these TLR9 agonists were high secretion of IFN-alpha and IP-10, the central cytokine and chemokine for activation of immune responses by TLR9 agonists. Two possible candidates were identified for further evaluation, EnanDIM-C and EnanDIM-A (Fig. 1d).

### EnanDIM® molecules activate components of innate and adaptive immune system

**Fig. 2:**
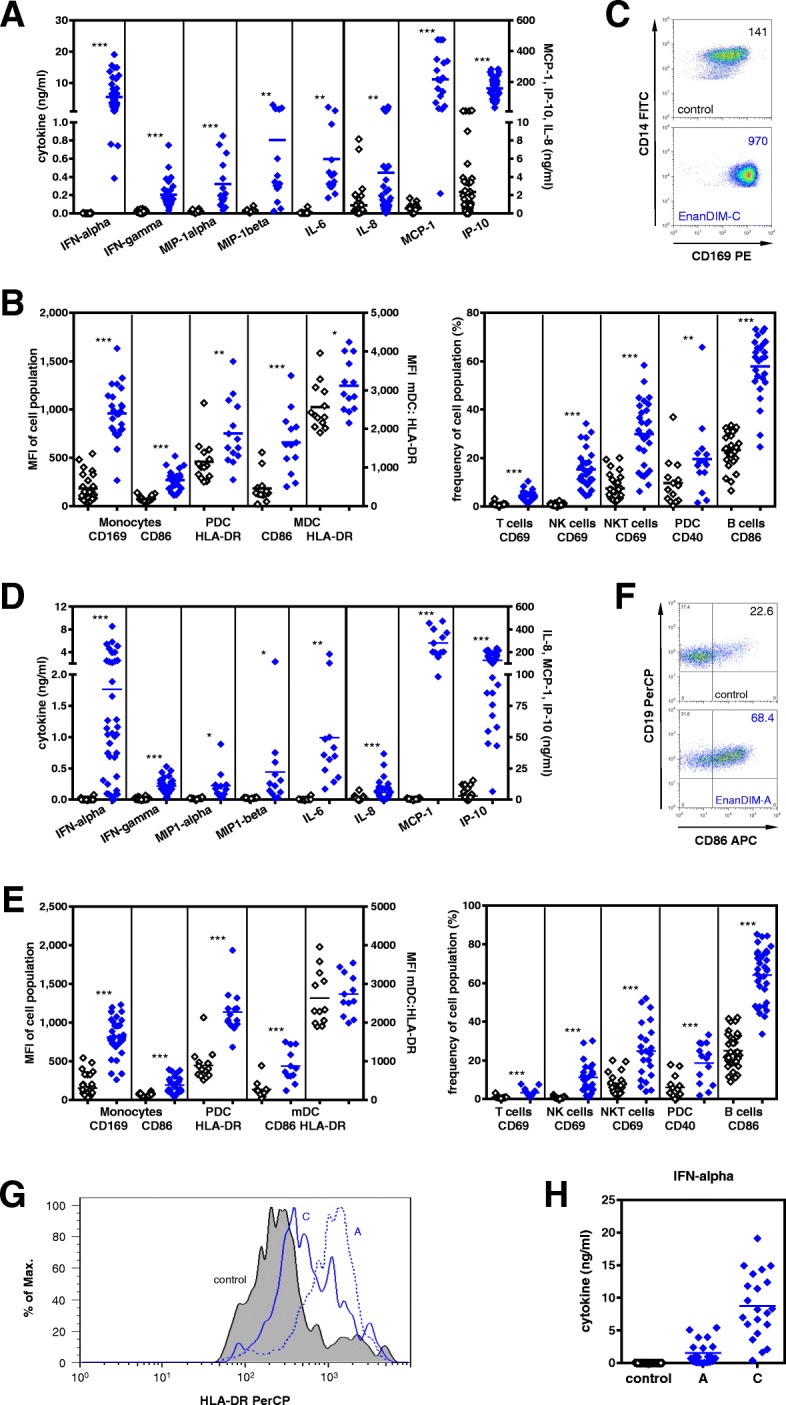
Immunological activation profile of EnanDIM-C (**a**-**c**), EnanDIM-A (**d**-**f**) and differences between both molecules (**g**, **h**). Human PBMC were treated without (black open squares) or with EnanDIM molecules (blue filled squares) at a final concentration of 3 μM for 48 h. Cytokines/chemokines were measured in cell culture supernatants (**a** *n* = 14–48, **d** *n* = 12–38, **h** *n* = 21**)** and activation of immune cells was analyzed by flow cytometry (**b** *n* = 13–29, **c**, **e** n = 12–34, **f**, **g**). Means are shown, differences between EnanDIM®-treated PBMC and controls were calculated using the paired t-test (*p < 0.05, **p < 0.01 ***p < 0.001) (**a**, **b**, **d**, **e**, **h**). Results from representative experiments are shown (**c** MFI of CD169 within monocytes is shown, **f** frequency of CD86 within B cells is shown, **g** HLA-DR expression of pDC)

Together with cell-cell interaction, secretion of chemo- and cytokines are important tools of the immune system to mount an anti-tumor response. Treatment of human PBMC with EnanDIM-C molecules resulted in a strong secretion of IFN-alpha, IP-10, MCP-1 and IFN-gamma (Fig. 2a). EnanDIM-C stimulates TLR9-positive pDC and B cells: however other immune relevant TLR9-negative cells within human PBMC, like myeloid dendritic cells (mDC), monocytes, natural killer (NK) cells, NKT cells and T cells, are likely activated via pDC-released IFN-alpha or via cell-cell contact with activated TLR9-positive cells (Fig. 2b, c). The broad activation of this spectrum of cell types indicates a strong induction of the innate and the adaptive immune systems. EnanDIM-A exhibited a comparable activation pattern targeting similar components of immune system (Fig. 2d-f). Despite this, each EnanDIM® molecule exhibits a unique pattern of immunomodulatory activity, with EnanDIM-C showing the highest secretion of IFN-alpha and EnanDIM-A with the strongest up-regulation of MHC class II on TLR9-bearing pDC (Fig. 2g, h).

### TLR9-specificity of EnanDIM® in vitro

**Fig. 3:**
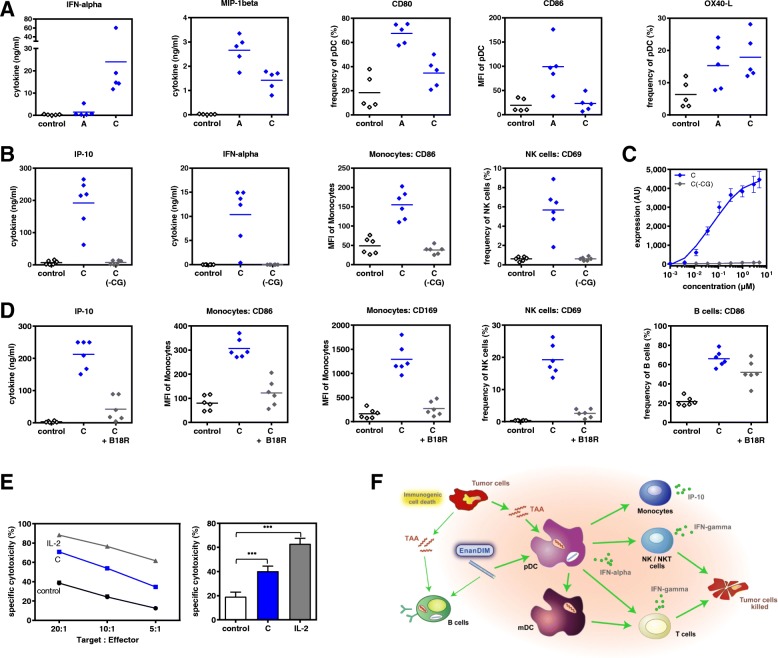
TLR9-specific effects of EnanDIM-C and EnanDIM-A in vitro. **a**, effect on pDC isolated from human PBMC: incubation with EnanDIM-A and EnanDIM-C at a final concentration of 3 μM for 48 h, analysis for cytokine / chemokine production and presence of activation markers, *n* = 5. **b**, **c**, CpG-dependency of EnanDIM-C effect: **b**, incubation of donor PBMC with EnanDIM-C or CG-depleted variant EnanDIM-C(-CG) at a final concentration of 3 μM for 48 h, analysis for cytokine / chemokine production and activation of cell surface markers on monocytes and NK cells, *n* = 6. **c**, incubation of ELAM41 reporter cells with the indicated concentrations of EnanDIM-C or EnanDIM-C(-CG) for 7 h and determination of the amount of fluorescent protein via flow cytometry. The expression level of eGFP is represented by the product of the relative amount of fluorescent cells and the fluorescence intensity (AU ± SEM) from 3 independent experiments. **d**, type-I interferon dependency of EnanDIM-C effect via IFN-alpha capture by B18R protein (vaccinia virus-encoded receptor with specificity to type I interferons): incubation of donor PBMC with EnanDIM-C at a final concentration of 3 μM ± B18R protein (final concentration 0.5 μg/ml) for 48 h, analysis for IP-10 production and activation of cell surface markers on monocytes, NK cells and B cells, n = 6. **e**, cytotoxic effect of EnanDIM-C in human PBMC against Jurkat cells: incubation of donor PBMC (*effector cells*) with EnanDIM-C at a final concentration of 3 μM for 48 h, co-culture of PBMC with Jurkat cells as *target cells* for 4 h, use of different *target:effector* ratios, quantification of *target cell* death by flow cytometry - shown is one representative donor (left) as well as mean values of 8 different donors ±SEM at a target:effector ratio of 10:1 (right), ***p < 0.001; One way ANOVA, Dunnett’s multiple comparisons test. **f**, proposed immunomodulatory mode-of-action of the TLR9 agonist EnanDIM®

To further investigate the mode-of-action of EnanDIM® molecules and their TLR9-specificity, the effect of EnanDIM-C and EnanDIM-A on isolated TLR9-positive pDC was compared. While EnanDIM-C induced a stronger IFN-alpha production by pDC, for EnanDIM-A a more pronounced increase of CD80 and CD86 surface marker expression on pDC was observed, confirming the preferential immune response pattern for each EnanDIM® molecule (Fig. 3a). The observed immune activation was strictly dependent on the presence of CG-motifs, known to be recognized by TLR9. Cytokine secretion and cellular activation was abrogated when a CG-depleted variant EnanDIM-C(-CG) was used (Fig. 3b). This was confirmed in the reporter cell line ELAM41 [26], where EnanDIM-C, but not EnanDIM-C(-CG), stimulated the TLR9-triggered NF-kappaB pathway indicated by expression of eGFP resulting in increased fluorescence (Fig. 3c). Furthermore, it was shown that an intact type I IFN pathway is crucial for the immunomodulatory effect of EnanDIM-C, since co-incubation with B18R protein (vaccinia virus-encoded receptor with binding capability to type I interferons) clearly reduced the activation of TLR9 negative cells and secretion of IP-10 (Fig. 3d). The reduction of B cell activation was less pronounced due to their TLR9 positivity allowing a direct stimulation.

### Cytotoxic activity of EnanDIM® in vitro

To provide evidence that stimulation of NK cells within human PBMC by EnanDIM® molecules convert them into effective tumor destroying cells, functional experiments to analyze NK cell-mediated cytotoxicity were performed. PBMC were stimulated with EnanDIM-C and subsequently co-cultured with Jurkat cells - a human T leukemic cell line - as target cells. Indeed, an increased death of target cells was observed, indicating the induction of NK cell mediated cytotoxicity (Fig. 3e).

Taken together, the data obtained from in vitro studies confirm the proposed mode-of-action of EnanDIM® molecules primarily targeting TLR9-positive cells and thus triggering subsequent broad innate and adaptive immune responses (Fig. 3f).

### Immunologic activity of EnanDIM® and lack of acute toxicity in vivo

**Fig. 4:**
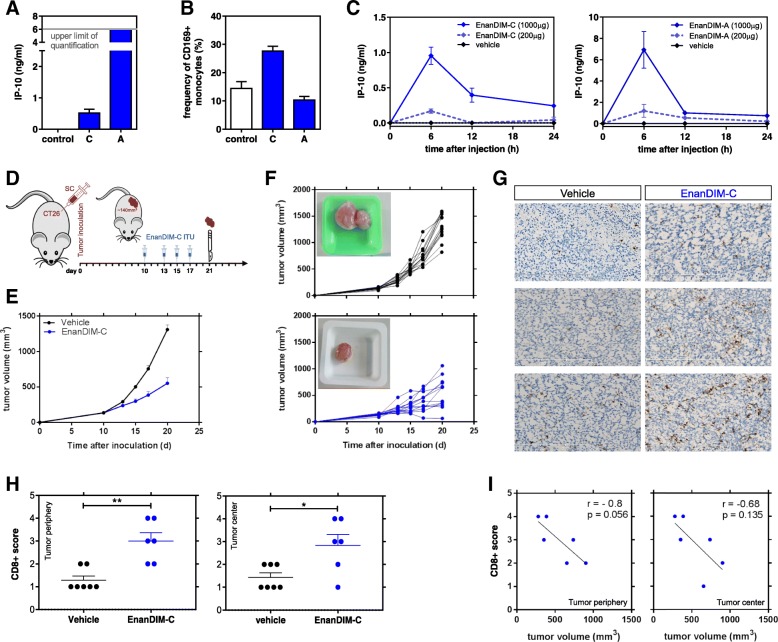
Immune activation and beneficial modulation of the TME by EnanDIM® molecules in vivo. S.c. injection of 10 mg EnanDIM-C, 50 mg EnanDIM-A or vehicle (0.9% NaCl) into female CD-1 mice and determination of IP-10 concentration (**a**) and frequency of CD169^+^ monocyte (CD11b + CD11c-) (**b**) after 24 h from blood samples. **c**, IP-10 serum levels measured at different time points after s.c. injection of indicated amounts of EnanDIM-C (left) or EnanDIM-A (right) into female Balb/c mice. **d-i**, impact of EnanDIM-C on the TME in vivo. **d**, Balb/c mice were inoculated s.c. with CT26 tumor cells. Established tumors (app. 140mm^3^, day 10) were injected with EnanDIM-C (i.tu.) or vehicle as control. Mice were sacrificed at day 21 for tumor preparation. **e**, mean tumor growth (+ SEM). **f**, tumor growth from individual mice (top: vehicle, bottom: EnanDIM-C) – inlay: example of tumor at sacrifice. **g**, examples of CD8^+^ staining (frozen sections). **h**, scoring of CD8^+^ cells in tumor periphery (**p ≤ 0.01, left) or tumor center (*p ≤ 0.05, right**)** from 7 mice per group, Mann-Whitney test was used. **i**, CD8^+^ T cell score vs tumor volume at day 21 in tumor periphery (left) or tumor center (right), correlation coefficients r and *p*-values from Pearson correlation analyses

EnanDIM® molecules were used in a maximum feasible dose (MFD) mouse study to evaluate their acute toxicity at very high single doses. EnanDIM-A was injected subcutaneously (s.c.) at 50 mg (app. 2000 mg/kg) and EnanDIM-C at 10 mg (app. 400 mg/kg). None of the EnanDIM® molecules led to mortality, clinical signs or body weight changes and macroscopic organ evaluation at day 15 revealed also no signs of toxicity (*data not shown*). In this model, treatment of mice with EnanDIM® molecules resulted in a clear peripheral immune activation represented by increased levels of IP-10 after 24 h (Fig. 4a). At the same time, up-regulation of CD169 on monocytes/macrophages was observed, however only for EnanDIM-C (Fig. 4b), while the strongly activated monocytes/macrophages in EnanDIM-A treated mice had likely already migrated into lymphoid tissues at the time of analysis [27]. As expected, the immune activation had subsided 15 d after the injection (*data not shown*). In line with this, an early dose-dependent increase of serum IP-10 level with a peak after approximately 6 h was visible both for EnanDIM-C and EnanDIM-A (Fig. 4c).

### Modulation of the TME by EnanDIM® in vivo

The presence of CD8^+^ T cells in the TME is a crucial pre-requisite for the success of immuno-oncological approaches. Given their mode-of-action EnanDIM® molecules should provide signals for recruitment of immune cells to the TME. EnanDIM-C was injected into established tumors in the CT26 colon carcinoma model (Fig. 4d). Tumor growth was significantly reduced in EnanDIM-C treated mice (Fig. 4e, f), and immunohistochemical analysis of tumors showed a significant increase of CD8^+^ T cells within the tumor (Fig. 4g, h). A trend towards a correlation of high CD8^+^ T cell numbers with small tumor volumes was also observed (Fig. 4i).

### Anti-tumor effects of EnanDIM® in syngeneic murine tumor models

**Fig. 5:**
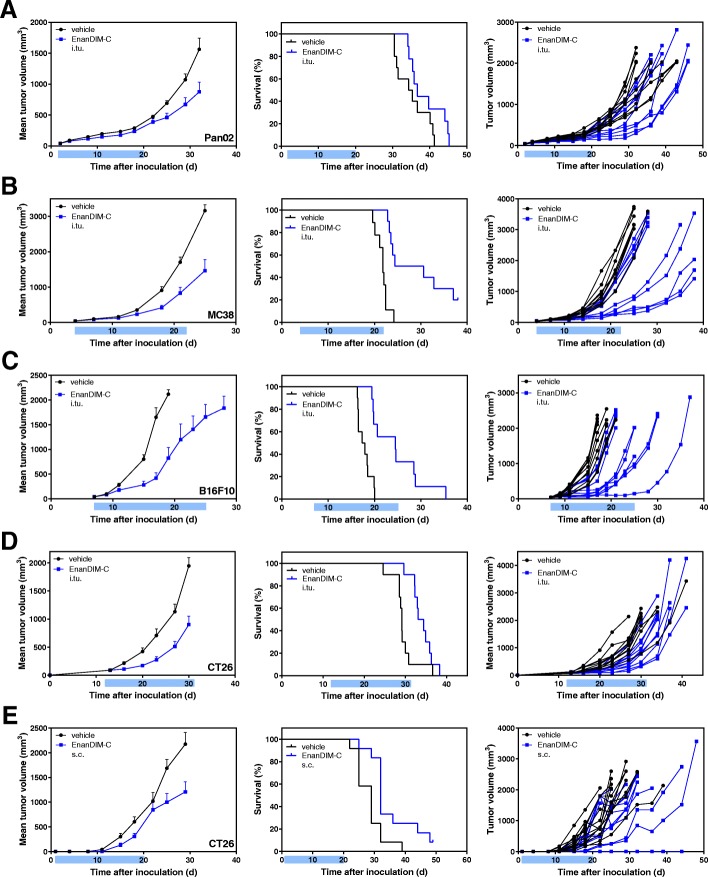
Anti-tumor effect of EnanDIM-C in various syngeneic tumor models. 10 mice per group were inoculated s.c. with either Pan02 pancreas (**a**), MC38 colon (**b**), B16F10 melanoma (**c**), or CT26 colon carcinoma (**d, e**) tumor cells. Established tumors (40–90 mm^3^, day 3 to day 13) were repeatedly injected with EnanDIM-C or vehicle (i.tu.) (**a-d**) or with EnanDIM-C or vehicle (s.c.) at the day after tumor inoculation (**e**). Mean tumor growth + SEM, Kaplan-Meier survival plots and individual tumor growth of all mice are shown from left to right. Mean tumor growth curves are continued until 50% of mice treated with vehicle have been sacrificed. Light blue bar on each x-axis indicates the treatment period. Log-rank analyses: MC38, *p* < 0.001; B16F10, *p* < 0.001; CT26 (i.tu), *p* < 0.05; CT26 (s.c.), *p* < 0.01

EnanDIM-C was next evaluated for its anti-tumor effects across a broad range of syngeneic murine tumor models, including Pan02 (pancreas carcinoma), MC38 (colon carcinoma), and B16F10 (melanoma). After s.c. inoculation of the respective tumor cells into Balb/c or C57BL/6 mice, EnanDIM-C (or vehicle) was injected multiple times into established tumors. The anti-tumor effect of EnanDIM-C varied with respect to tumor models ranking from low (Pan02) to clear (MC38, B16F10) reduction of tumor growth and, consequently, prolongation of survival (Fig. 5a-c). In order to evaluate a route of administration intended for a broader potential clinical application, EnanDIM-C was injected systemically (s.c.) in the CT26 model which showed a comparable anti-tumor effect to local (i.tu.) injection in this model (Fig. 5d, e).

### Long-lasting immune memory through EnanDIM® in EMT-6 and A20 murine tumor models

**Fig. 6:**
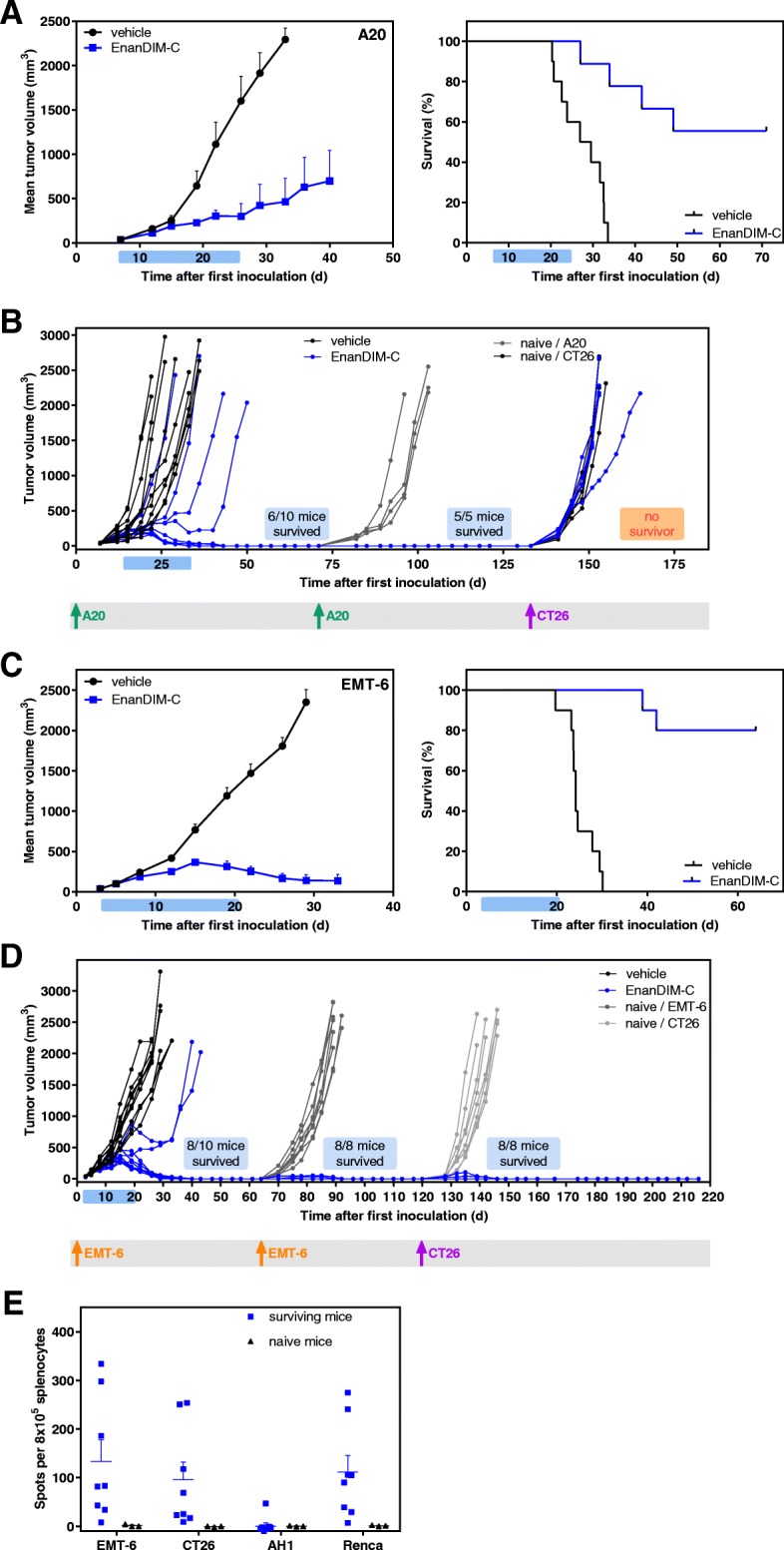
Persistent immunological anti-tumor memory induced by EnanDIM-C in two syngeneic tumor models. 10 mice per group were inoculated s.c. with either A20 lymphoma (**a, b**), or EMT-6 breast cancer (**c, d**) tumor cells. Established tumors (40 mm^3^, day 3 to day 7) were injected with EnanDIM-C or vehicle (i.tu.). Mean tumor growth + SEM (left), and Kaplan-Meier survival plots (right) are shown (log-rank analyses: A20, *p* < 0.0001; EMT-6, *p* < 0.0001). Mean tumor growth curves are continued until 50% of mice treated with vehicle have been sacrificed. Light blue bar on each x axis indicates the treatment period. **b**, surviving mice from the A20 tumor model as well as age-matched naïve mice were re-challenged with A20 cells (1st re-challenge) and surviving mice were subsequently re-challenged with CT26 cell (2nd re-challenge) as specified. **d**, surviving mice from the EMT-6 tumor model as well as age-matched naïve mice were re-challenged with EMT-6 cells (1st re-challenge) and surviving mice were subsequently re-challenged with CT26 cell (2nd re-challenge) as specified. Individual tumor growth of all mice is shown and respective tumor cell injections are marked. **e**, spleen cells from naïve mice and from mice surviving two EMT-6 and one CT26 tumor inoculation were collected, co-cultured with either EMT-6, CT26 cells, AH1 peptide or Renca cells and subjected to ELISpot assay to quantify the IFN-gamma secreting cells

Treatment of mice with EnanDIM-C in the syngeneic A20 lymphoma model showed substantial tumor growth inhibition (TGI) of 78% and a highly significant increase of survival (Fig. 6a). In fact, in six out of ten mice the tumors completely disappeared. Five surviving mice were subsequently subjected to a re-challenge with A20 cells without any further treatment. All five mice completely rejected the second inoculation of A20 cells, in contrast to age-matched naïve mice, indicating that clearance of the initial lymphoma following EnanDIM-C treatment resulted in the formation of protective anti-tumor memory. However, re-challenge of surviving mice with CT26 tumor cells led to tumor growth in all mice indicating no cross-reaction of the induced immunity (Fig. 6b). EnanDIM-C induced a profound anti-tumor effect in the syngeneic EMT-6 breast cancer model with a substantially inhibited tumor growth (TGI: 85%) and a significantly augmented survival (Fig. 6c). Notably, eight out of ten mice showed complete regression of tumor and re-challenge of the surviving mice with EMT-6 cells led to tumor-free survival of all of them, which was in contrast to age-matched naïve mice. Again, EnanDIM-C induced a sustained anti-tumor immune memory against EMT-6 cells. Unexpectedly, re-challenge of surviving mice with CT26 tumor cells led to tumor rejection in all surviving mice indicating a cross-reactive immunity between antigens expressed by EMT-6 and CT26 cells (Fig. 6d). This was confirmed by an ELISpot assay, showing a significantly increased number of IFN-gamma secreting cells after re-stimulation with EMT-6 or CT26 tumor cells as well as Renca cells compared to naïve mice (Fig. 6e).

## Discussion

EnanDIM® molecules constitute a novel family of L-nucleotide-protected TLR9 agonists. They induce a broad stimulation of cells involved in innate and also adaptive immune responses, with pDC and B cells as primary and mDC, monocytes NK-, NKT cells and T cells as secondary target cells. Together with their elicited chemokines/cytokines these TLR9-agonists play crucial roles in the body’s anti-tumor immune response: IFN-alpha stimulates several key regulatory immune cells and thereby initiates innate and also adaptive immune responses [28], the latter especially by activating CD8-alpha^+^ dendritic cells able to cross-present antigens to cytotoxic T cells [29, 30]. The chemokine IP-10 attracts activated T and NK cells and also has angiostatic potential [31, 32]. IFN-gamma may be secreted by NK cells in response and is one of several mediators for a TH1 immune response [33].

Notably, the secretion of the pro-inflammatory and angiogenic cytokine IL-8 was only moderately induced by EnanDIM® and considerably less when compared with other TLR9 agonists (Additional file 1: Figure S1). Strong IL-8 secretion induced by class B CpG-ODN containing a complete PTO backbone was independent from TLR9-binding CG-motifs [34]. Generally, off-target immunological effects on certain immune cell populations usually caused by PTO-modifications in CpG-ODN are non-detectable with EnanDIM-C. This was supported by the absence of toxicities in a MFD study using EnanDIM-C and EnanDIM-A at very high dose levels in vivo which contrasts to previous publications describing side effects for PTO-modified CpG-ODN [16–18]. Taken together, EnanDIM-C/−A show a beneficial immune profile and initial data from the MFD study may predict an absence of toxicities and severe adverse events in subsequent clinical development.

EnanDIM-C treatment led to a recruitment of CD8^+^ T cells to the TME in vivo, which can be explained by the primary activation of pDC to induce secretion of IFN-alpha synergizing with the secondary induction of IFN-gamma for the secretion of IP-10 from monocytes [35]. Effector CD8^+^ T cells, TH1 cells and NK cells express CXC-chemokine receptor 3 (CXCR3), which is the receptor for the TH1-type chemokines CXC-chemokine ligand 9 (CXCL9) and IP-10 (CXCL10). These cells can migrate into tumors in response to these chemokines [32, 36, 37], thereby increasing the number of T cells in the tumor. The presence of a T cell-inflamed TME in so-called “hot tumors” is linked with improved responses to cancer immunotherapies including checkpoint inhibitors [38, 39].

We have shown that the modulation of the TME, shown as CD8+ T cell infiltration, is associated with a reduction of tumor growth in the CT26 colon carcinoma model. The anti-tumor effect was also observed for other tumor models and consequently resulted in an improved survival of mice. More importantly, treatment with EnanDIM-C resulted in a complete tumor regression in the majority of mice in A20 lymphoma or EMT-6 breast cancer models and all surviving mice rejected tumor cells in a re-challenge study, suggesting a sustained immune memory against the tumor. The complete regression of established tumors in the EMT-6 model is especially remarkable, since therapeutic blockade of PD-L1 alone had little or no effect in this model [40]. EMT-6 is known for its immune excluded TME and only a combination of blockade of PD-L1 and TGF-beta resulted in a pronounced anti-tumor effect together with an infiltration of T cells [40]. Furthermore, complete tumor rejection of a secondary CT26 tumor may indicate cross-reactivity against shared antigens between different tumor types, confirmed by ELISpot responses not only against EMT-6 but CT-26 and even Renca cells. Anti-tumor efficacy of EnanDIM-C varied between different syngeneic models indicating different tumor properties in responding to immune modulation. It is well known that syngeneic models differ in their immunogenicity and in their ability to respond to immuno-oncological approaches including checkpoint inhibitors: Analyses of syngeneic murine tumor models revealed strong differences in mutational load, type of mutations, gene expression in immune-related pathways as well as composition and magnitude of the tumor immune infiltrates [41]. Immunosuppressive cell types in the TME dominated in tumor models that did not respond to immune-checkpoint blockade, whereas cytotoxic effector immune cells were enriched in responsive models.

The described immunological features of EnanDIM® molecules, their potent anti-tumor responses and the lack of off-target effects renders them as an ideal combination partner for other immunotherapeutic approaches. Especially the fact, that the mode-of-action of EnanDIM® molecules via TLR9 starts upstream of the targets of checkpoint inhibitors like anti-PD-1/anti-PD-L1 a combinatory approach may ideally be suited for a synergistic immune activation and thus enhanced anti-tumor effects. This was currently shown by PTO-modified TLR9 agonists in mouse tumor models [42, 43] and also in a clinical trial in advanced melanoma [44].

**Fig. 7:**
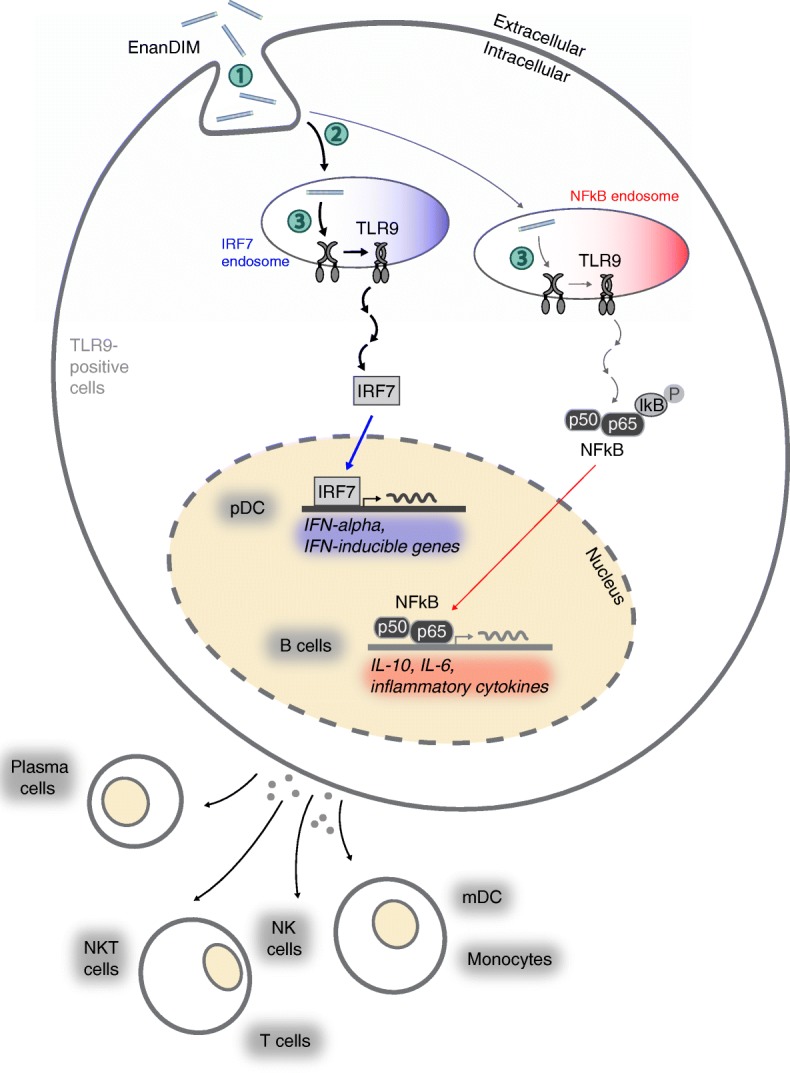
Mode-of-action of EnanDIM® molecules: Differential activation of secondary cells. Variation of sequences and thus secondary conformation have an influence on ① unspecific DNA-uptake by TLR9-positive cells, ② differential uptake into the respective (early or late) endosomes resulting in either IRF7 or NF-kappaB activation followed by induction of specific cytokine pattern and surface marker expression resulting in differential activation of (TLR9-negative) cell subpopulations, and ③ specific binding to the TLR9 and thus strength of the response and subsequent thresholds for cell activation (which may differ between cell types)

A major question regarding the use of EnanDIM® molecules with different sequences is how these TLR9 agonists are able to induce different immunological responses in the same donor PBMC when only one receptor, TLR9, is involved. A possible explanation for this phenomenon is that molecules with distinct sequences may exhibit differences in molecule uptake, intracellular distribution and receptor binding [45] (Fig. 7). The distribution into two distinct types of endosomes results in the induction of two specific signaling pathways: a) activation of NF-kappaB inducing the production of pro-inflammatory cytokines and acquisition of antigen-presenting function, and b) activation of interferon regulatory factor 7 (IRF7) leading to type I IFN (e.g. IFN-alpha) production [46, 47], which is crucial to link the stimulated innate response to the adaptive arm of the immune system [28]. This way differential mixtures of IFN-alpha and other cytokines as well as the induction of specific surface molecules in pDC determine the subsequent characteristic activation of secondary target cells such as NK cells, monocytes and mDC. Our own data regarding IFN-alpha secretion of pDC, target specificity for TLR9 and the dependency of a broad immune activation by EnanDIM® molecules on an intact type I IFN pathway support the relevance of the published signaling pathway of TLR9 agonists [46, 47] for EnanDIM®.

As described, EnanDIM-C is a potent inducer of the IFN-alpha pathway resulting in a strong activation of TLR9-negative cells, like NK cells and monocytes but less pronounced B cell activation. However, with EnanDIM-B we identified an EnanDIM® molecule mainly triggering the B cell pathway (Additional file 2: Figure S2). This way it will be possible to broaden the spectrum of possible applications for the EnanDIM® family.

## Conclusion

EnanDIM® molecules activate the innate and adaptive immune system. Their mode-of-action is strictly dependent on presence of CG-motifs specifically targeting TLR9. Off-target effects are avoided due to the lack of chemical modifications and a wide therapeutic window may thus be enabled. The immunological and resulting anti-tumor potential of EnanDIM®, including beneficial TME modulation, inhibition of tumor growth and induction of long-lasting tumor-specific memory, favors the EnanDIM® family of TLR9 agonists for further preclinical and clinical development as cancer immunotherapy for systemic and local administration.

## Additional files

Additional file 1: **Figure S1.** Immunological activation profile of EnanDIM-C in comparison to the common PTO-modified CpG-ODN CpG 7909 (ProMune). In vitro stimulation of human PBMC cells at the indicated concentrations (**a**, **b**) or a final concentration of 3 μM (**c**) and read-out after 48 h. **a**, dose-dependent stimulation of cytokine/chemokine production: anti-tumor IFN-alpha (top left), anti-angiogenic IP-10 (top right), IFN-gamma (bottom left), unfavorable IL-8 (bottom right), *n* = 5. **b**, dose-dependent activation of cell surface marker CD86 on monocytes (left, *n* = 4), CD86 on B cells (right, n = 5). **c**, activation of cell surface marker CD69 on NK cells (left), CD69 on T cells (right), *n* = 20. Values obtained after activation with different concentrations of both TLR9 agonists were normalized to the values obtained after activation with EnanDIM-C at a final concentration of 3 μM. (PPTX 102 kb)

Additional file 2: **Figure S2.** Selection of an EnanDIM® molecule with strong B cell activation. **a**, screening for increase of B cell activation (CD86 + CD19+): incubation of human PBMC with EnanDIM® molecules as well as a reference molecule at a final concentration of 3 μM for 48 h in vitro. Expression of CD86 on monocytes and B cells was quantified and normalized to the reference molecule (N ranges from 2 to 29 for the different molecules). EnanDIM-A, -B and -C are shown as black solid circles. **b**, dose-response curves of human PBMC stimulation in vitro. PBMC were stimulated with EnanDIM-B at the indicated concentrations for 48 h, cytokines (*n* = 8) and cellular activation markers (n = 5) were analyzed. (PPTX 72 kb)

## Abbreviations

eGFP: Enhanced green fluorescent protein
i.tu: Intratumorally
IHC: Immunohistochemistry
IP-10: Interferon-inducible protein 10
MCP-1: monocyte chemoattractant protein-1
mDC: Myeloid dendritic cells
MFD: Maximum feasible dose
MFI: Mean fluorescence intensity
NF-kappaB: Nuclear factor kappa-light-chain-enhancer of activated B-cells
NK: Natural killer
ODN: Oligodeoxynucleotides
PAMP: Pathogen-associated molecular patterns
PBMC: Peripheral blood mononuclear cells
pDC: Plasmacytoid dendritic cells
PO: Phosphodiester
PRR: Pattern recognition receptor
PTO: Phosphorothioate
s.c.: Subcutaneously
TGI: Tumor growth inhibition
TLR: Toll-like receptor
TME: Tumor microenvironment
