# Supplementary figures and images for: EnanDIM - a novel family of L-nucleotide-protected TLR9 agonists for cancer immunotherapy
Source: J Immunother Cancer. 2019 Jan 8;7:5. doi: 10.1186/s40425-018-0470-3 (PMC6323716; doi:10.1186/s40425-018-0470-3)

## Slide 1
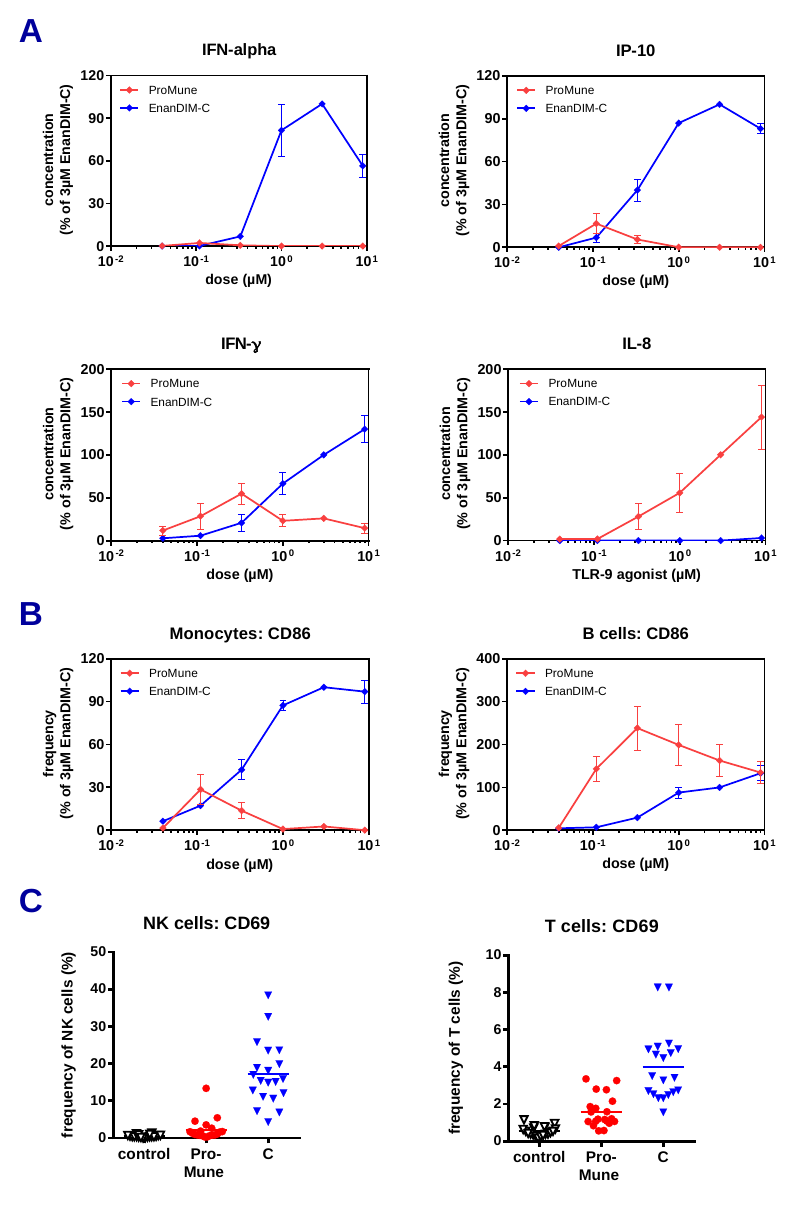

A
B
C

Supplement: Supplementary file 1 — Figure S1. Immunological activation profile of EnanDIM-C in comparison to the common PTO-modified CpG-ODN CpG 7909 (ProMune). In vitro stimulation of human PBMC cells at the indicated concentrations (a, b) or a final concentration of 3 μM (c) and read-out after 48 h. a, dose-dependent stimulation of cytokine/chemokine production: anti-tumor IFN-alpha (top left), anti-angiogenic IP-10 (top right), IFN-gamma (bottom left), unfavorable IL-8 (bottom right), n = 5. b, dose-dependent activation of cell surface marker CD86 on monocytes (left, n = 4), CD86 on B cells (right, n = 5). c, activation of cell surface marker CD69 on NK cells (left), CD69 on T cells (right), n = 20. Values obtained after activation with different concentrations of both TLR9 agonists were normalized to the values obtained after activation with EnanDIM-C at a final concentration of 3 μM. (PPTX 102 kb) [file 40425_2018_470_MOESM1_ESM.pptx]

## Slide 1
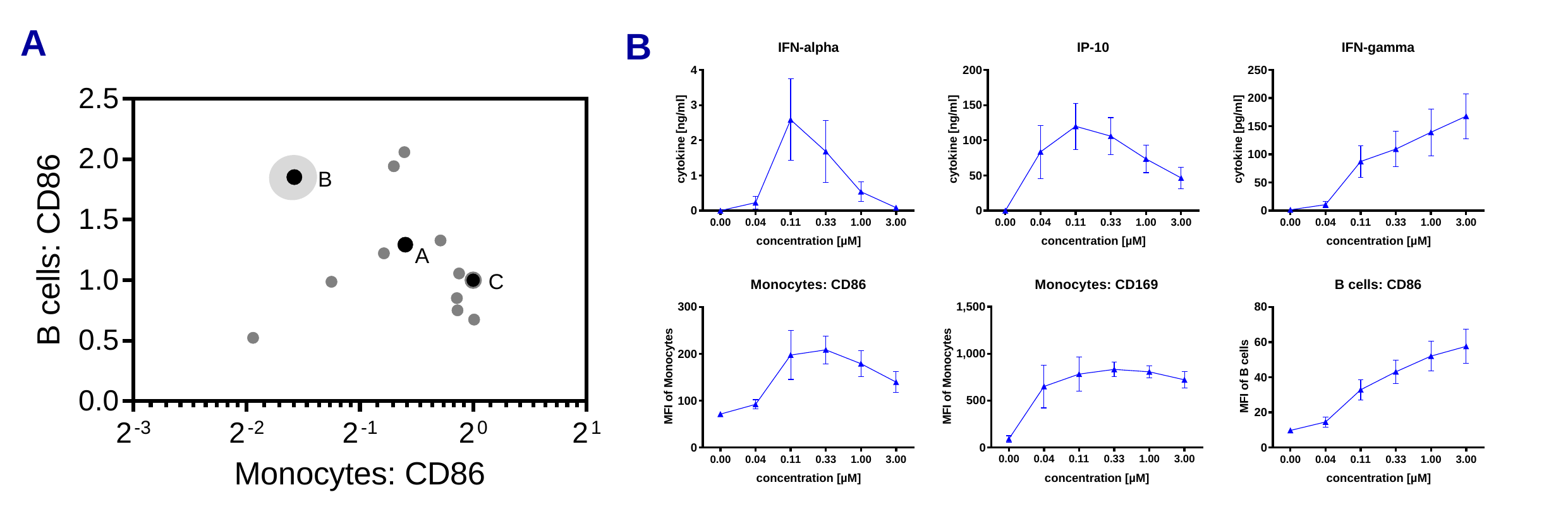

A
B

Supplement: Supplementary file 2 — Figure S2. Selection of an EnanDIM® molecule with strong B cell activation. a, screening for increase of B cell activation (CD86 + CD19+): incubation of human PBMC with EnanDIM® molecules as well as a reference molecule at a final concentration of 3 μM for 48 h in vitro. Expression of CD86 on monocytes and B cells was quantified and normalized to the reference molecule (N ranges from 2 to 29 for the different molecules). EnanDIM-A, -B and -C are shown as black solid circles. b, dose-response curves of human PBMC stimulation in vitro. PBMC were stimulated with EnanDIM-B at the indicated concentrations for 48 h, cytokines (n = 8) and cellular activation markers (n = 5) were analyzed. (PPTX 72 kb) [file 40425_2018_470_MOESM2_ESM.pptx]
